# αA-Crystallin Peptide ^66^ SDRDKFVIFLDVKHF ^80^ Accumulating in Aging Lens Impairs the Function of α-Crystallin and Induces Lens Protein Aggregation

**DOI:** 10.1371/journal.pone.0019291

**Published:** 2011-04-28

**Authors:** Puttur Santhoshkumar, Murugesan Raju, K. Krishna Sharma

**Affiliations:** 1 Department of Ophthalmology, University of Missouri–Columbia School of Medicine, Columbia, Missouri, United States of America; 2 Department of Biochemistry, University of Missouri–Columbia School of Medicine, Columbia, Missouri, United States of America; University of Arkansas for Medical Sciences, United States of America

## Abstract

**Background:**

The eye lens is composed of fiber cells that are filled with α-, β- and γ-crystallins. The primary function of crystallins is to maintain the clarity of the lens through ordered interactions as well as through the chaperone-like function of α-crystallin. With aging, the chaperone function of α-crystallin decreases, with the concomitant accumulation of water-insoluble, light-scattering oligomers and crystallin-derived peptides. The role of crystallin-derived peptides in age-related lens protein aggregation and insolubilization is not understood.

**Methodology/Principal Findings:**

We found that αA-crystallin-derived peptide, ^66^ SDRDKFVIFLDVKHF ^80^, which accumulates in the aging lens, can inhibit the chaperone activity of α-crystallin and cause aggregation and precipitation of lens crystallins. Age-related change in the concentration of αA-(66-80) peptide was estimated by mass spectrometry. The interaction of the peptide with native crystallin was studied by multi-angle light scattering and fluorescence methods. High molar ratios of peptide-to-crystallin were favourable for aggregation and precipitation. Time-lapse recordings showed that, in the presence of αA-(66-80) peptide, α-crystallin aggregates and functions as a nucleus for protein aggregation, attracting aggregation of additional α-, β- and γ-crystallins. Additionally, the αA-(66-80) peptide shares the principal properties of amyloid peptides, such as β-sheet structure and fibril formation.

**Conclusions/Significance:**

These results suggest that crystallin-derived peptides such as αA-(66-80), generated *in vivo*, can induce age-related lens changes by disrupting the structure and organization of crystallins, leading to their insolubilization. The accumulation of such peptides in aging lenses may explain a novel mechanism for age-related crystallin aggregation and cataractogenesis.

## Introduction

The lens is endowed with highly stable, long-lived proteins known as crystallins. Three lens proteins—α-, β- and γ-crystallins—account for nearly 90 percent of the total lens proteins and have both a structural and a refractive role [1]. The chaperone activity of the α-crystallin subunits, αA- and αB-crystallin, is believed to be integral to the maintenance of lens transparency [2], [3]. Aggregates of crystallin proteins begin to form during aging, and the lens begins to accumulate modified proteins. With aging, crystallins slowly lose their protective mechanisms for maintaining lens clarity and function [4], [5], leading to deterioration of the optical quality of the lens and, eventually, to the development of age-onset cataract.

The human lens has both water-soluble (WS) and water-insoluble (WI) fractions that contain protein aggregates composed of modified and C-terminally truncated αA- and αB-crystallins, crystallin fragments, and complexes of α–β–γ crystallins [6]–[8]. Compared to young human lenses, aged and cataract human lenses have significantly increased quantities of crystallin fragments [9]–[11]. The nuclear region of aged and cataract lenses has the highest percentage of WI proteins [6], and proteins in the nuclear region also exhibit the highest degree of age-related modifications (including truncations) and light-scattering properties [12], [13]. α-Crystallin in aged and cataract lenses also has diminished chaperone activity [3], [14], [15]. While it has long been known that in >40-year-old lenses, almost all of the α-crystallin in the nuclear region is in the WI fraction [16], [17], the reasons for the preferential insolubilization of α-crystallin in the nuclear region are not understood. That α-crystallin aggregation is a contributing factor in the development of lens opacity is further supported by the recent observation in the lens nuclear region of a significant decrease in the α-crystallin index (the percentage of scatter from small particles, representing mostly unbound α-crystallin) and its association with increasing nuclear opacity grades [18]. There is a strong correlation between the degree of post-translational modification and the degree of crystallin aggregation and insolubilization but it is yet to be demonstrated whether even the major modification-deamidation of crystallins [19] can cause the selective α-crystallin aggregation and insolubilization that occur in aging lenses [16].

In a previous study, we found that over 25 peptides are present in aged and cataract human lenses [10]. Among these peptides, we found that the αA-(66-80) peptide and its truncated forms αA-(66-75), αA-(67-80), and αA-(67-75) carry a portion of the αA-crystallin chaperone site [20]. αA-(66-80) and its truncated forms arise from the β3-strand of native αA-crystallin [21]. The αA-(66-80) peptide possesses sequence similarity to a region responsible for fibril formation in β-amyloid [22]. Previous studies have shown that β-amyloid peptide induces aggregation of a number of proteins [23]. We therefore hypothesized that peptides arising from the αA-66-80 region during aging might display properties similar to those of β-amyloid and, hence, might play a pivotal role in age-related crystallin aggregation. Studies have shown that peptides with a propensity to form fibril-like structures aggregate themselves and also aggregate with other proteins [24]. In the present study, we show that interactions between α-crystallin–derived peptide[s] and α-crystallin are sufficient to form high molecular weight (HMW) aggregates similar to those found in the nuclear region of human lenses during aging and cataract formation. Further, we show that intact α-, β- or γ-crystallins can be recruited to the insoluble complexes formed by the interaction between the crystallin-derived peptide and α-crystallin, thus providing the first clue to the molecular mechanisms of age-related crystallin aggregation and cataract formation.

## Results

### αA-Crystallin–derived αA-(66-80) peptide (SDRDKFVIFLDVKHF) distribution in the human lens

**Figure 1:**
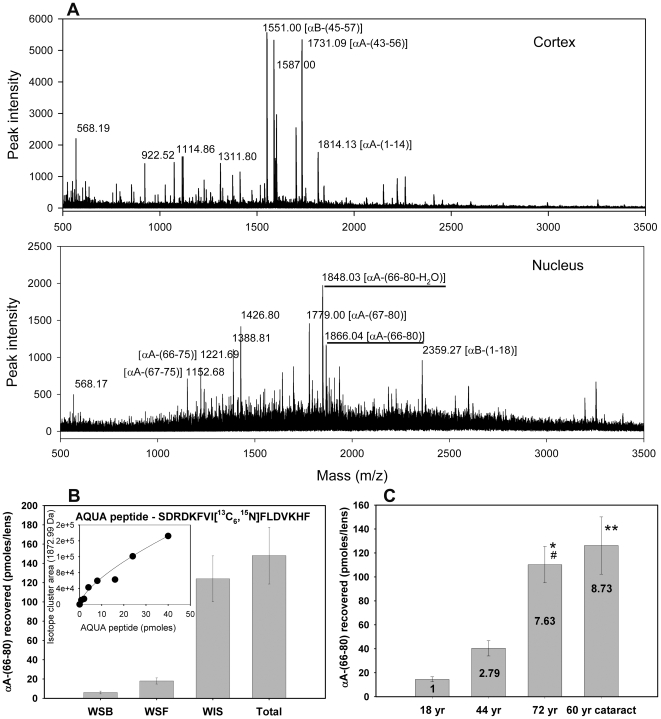
Distribution of the αA-(66-80) peptide in the lens. (A) MALDI qTOF MS/MS analysis of LMW peptides isolated from the cortex and nucleus of a >70-year-old human lens as described under methods. The αA-(66-80) peptide characterized in this study is highlighted in the panel labeled – nucleus. (B) αA-(66-80) peptide concentrations in a >70-year-old lens fractions (WSB, water-soluble bound; WSF, water-soluble free; WIS, water-insoluble sonicated fractions). The amount of the peptide was estimated using ^13^C ^15^N-labelled spiked synthetic peptide standards (AQUA peptide) during peptide isolation. The inset figure is the standard curve for the isotope cluster area of the AQUA peptide used to determine *in vivo* peptide concentration. (C) Concentrations of αA-(66-80) peptide in human lenses of 18-, 44-, 60- and 72-year age groups. Lenses that were 60 years old were cataract lenses. Values within the bar show the multiple increased concentrations as compared to the 18-year-old lens. The values are mean ± SD of three analyses. * P<0.001 compared to 18 year, # P<0.01 compared to 44 year, ** P<0.001 compared to 18 and 44 year. P value determined using ANOVA. These results show an age-dependent increase in the concentration of αA-(66-80) peptide in human lenses and these peptides are associated with the WIS fraction. The cataract lenses show a higher level of αA-(66-80) peptide than the expected level of the peptide in age-matched non-cataract lenses.

We previously showed that a number of peptides and protein fragments derived from crystallins accumulate in aged and cataractous human lenses [10]. Among these peptides, αA-(66-80) peptide and truncated forms of this peptide were conspicuous because they contained residues that contributed to the chaperone site we had previously identified in αA-crystallin [20]. To determine the spatial distribution of the αA-(66-80) peptide in >70-year-old human lenses, we isolated the peptides from the cortical and nuclear extracts and subjected them to MALDI qTOF MS analysis. We found that the αA-(66-80) peptide is present only in the nuclear region of the >70-year-old lens (Figure 1A). The temporal concentration of the αA-(66-80) peptide was estimated using a C^13^N^15^-labeled αA-(66-80) peptide as an internal standard in MALDI qTOF MS analysis [25]. The αA-(66-80) peptide concentration increased with age, and its concentration was highest in cataract lenses (Figure 1C). Additionally, the αA-(66-80) peptide was maximally associated with WIS crystallins (Figure 1B). The WIS-to-WS ratio was 10.0 for αA-(66-80) peptide in the >70-year-old human lenses. The amount of αA-(66-80) peptide present in >70-year-old human lenses was 2.33±0.6 n mol per g lens tissue. The WIS fraction of >70-year-old human lens was estimated to contain 0.55±0.03 nmol αA-(66-75), 1.4±0.1 nmol of αA-(67-80), and 0.33±0.02 nmol αA-(67-75) per g lens tissue, all rising from 66-80 region of αA-crystallin. Since previous studies have shown that peptides that interact with other proteins are generally recovered at diminished levels from a protein-rich media [26], it is likely that the solubilization and extraction procedure we used did not recover all the peptides present in >70-year-old lens. Therefore, the actual amount of αA-(66-80) peptide in aged and cataract lenses could be even higher than the amount we documented in this study.

### αA-(66-80) Peptide and aggregation and precipitation of α-crystallin

**Figure 2:**
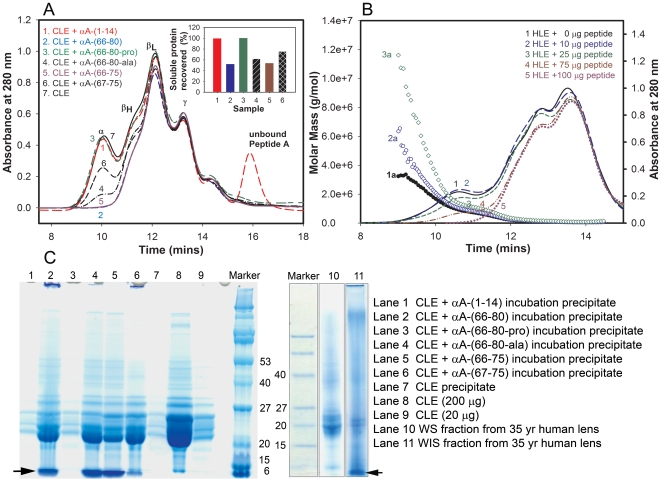
The αA-(66-80) peptide–induced aggregation and precipitation of crystallins. (A) Size-exclusion column elution profiles of soluble crystallins from WS CLE (200 µg) following incubation with or without different synthetic peptides (100 µg) for 16 hrs at 37°C. The chromatography was performed using a TSK G5000PW_XL_ column (7.8 mm×30 cm) and the elution was monitored using a 280 nm absorption detector. The inset in the figure shows the amount of soluble protein recovered after peptide treatment and centrifugation at 3000 rpm for 10 min. Samples 1-7 in the inset correspond to samples 1-7 analyzed by the size-exclusion column. The results show that the effect of αA-(66-80) peptide is sequence specific. The addition of αA-(66-80), αA-(66-75) and αA-(67-75) peptides, present in human lens WIS fractions, causes precipitation of crystallins when incubated with CLE. (B) Effect of the peptide on 35-year-old human lens crystallins. HLE (190 µg) was incubated with various amounts of synthetic αA-(66-80) peptide for 16 h at 37°C. The samples were centrifuged, and the supernatants were chromatographed on a TSK G5000PW_XL_ column connected to a multi-angle light scattering instrument (Wyatt Technology). Lines 1a, 2a and 3a show the distribution of molecular mass across the UV profile of samples 1, 2 and 3, respectively. Mass profiles of samples 4 and 5 could not be analyzed due to instrument limitations. The results show that the presence of increased amounts of αA-(66-80) peptide in the incubation mixture leads to increased aggregation, light scattering and precipitation. (C) SDS-PAGE analysis of precipitates of CLE and αA-derived peptides or substituted αA-peptides from panel A and the WS and WIS fractions from a 35-year-old human lens. The left arrow on lane 1 points to the precipitation of test peptides with crystallins and the right arrow on lane 11 points to the presence of peptides in human WIS fraction. The results of SDS-PAGE analysis show the precipitate formed when αA-crystallin-derived peptides are incubated with CLE that contains both the lens crystallins and the peptide tested. The data also confirm the presence of LMW protein fragments in the WIS fraction from human lenses.

Earlier we reported that *in vitro*–generated peptides from oxidized βL-crystallins facilitate the aggregation of denaturing proteins [27]. Because the bulk of αA-(66-80) peptide was found in the WIS fraction of aged and cataract lenses, we hypothesized that this peptide may have a role in insolubilization of α-crystallin in the nuclear region of lenses. To test our hypothesis *in vitro*, we added synthetic αA-(66-80) peptide to the WS protein fraction of calf lens extract (CLE, <1 yr) and of 35-year-old human lens extract (HLE), prepared in 50 mM phosphate buffer (pH 7.2) and incubated for 16 h. Lens proteins from this age group of human lenses were chosen because they represent proteins prior to complete insolubilization in the nuclear region [16] and are less modified than the crystallins from lenses older than 50 years, which have increased concentrations of insoluble α-crystallin in the nuclear region. Wide-ranging peptide-to-crystallin ratios (1∶1.9 to 1∶19, w/w) were used to determine the concentration-dependent effect of peptide on preferential insolubilization of α-crystallin present in lens extracts. In both CLE and HLE, the addition of the αA-(66-80) peptide led to a decrease in α-crystallin in the soluble fraction (Figure 2A and 2B). The decrease was directly proportional to the amount of peptide added as shown in Figure 2B with HLE and αA-(66-80) peptide. As we found with αA-(66-80) added to HLE (Figure 2B), the addition of increasing amounts of αA-(66-80) peptide to CLE resulted in the precipitation and removal of increasing quantities of α-crystallin (Figure S1). More than 50% of the soluble α-crystallin from 200 µg of CLE precipitated following incubation with 50 µg of αA-(66-80) peptide (Figure S1). The peptide also caused an increase in the molecular mass of the lens proteins that remained soluble (Figure 2B). SDS-PAGE analysis showed that the precipitate formed in CLE after the addition of αA-(66-80) peptide was composed mainly of α-crystallin and the αA-(66-80) peptide (Figure 2C, lane 2). SDS-PAGE analysis of WIS fraction in HLE from a 35-year-old human lens showed low molecular weight (LMW) peptides (arrow, Figure 2C) and crystallins and modified proteins (Figure 2C, lane 11), whereas the WS fraction from the same lens showed mainly proteins ∼20 kDa and higher (Figure 2C, lane 10).

The protein elution profiles in Figure 2A also show the effects in CLE of αA-(66-80) variants found in aged lenses and the effects of Pro- and Ala-substituted αA-(66-80) peptides, as compared to the control peptide αA-(1-14) (called peptide A in Figure 2A). The addition of peptides αA-(66-75), αA-(67-75) and αA-(66-80) to CLE resulted in maximum precipitation (Figure 2C), whereas the addition of Pro-substituted αA66-80 peptide (SDRDKF**P**IFLDVKHF) to CLE led to the least amount of precipitation, comparable to the amount of precipitation with αA-(1-14) peptide (Figure 2C and 2A inset). Replacing one of the hydrophobic residues, Val, with less hydrophobic Ala decreased precipitation of α-crystallin from CLE. Table 1 lists the tested peptides and the relative effectiveness of the peptides in precipitating crystallins *in vitro* during incubation. The peptide-to-crystallin monomer ratio was 1∶8 (mol/mol). Similar to the observations made with CLE, both αA-(66-80) and αA-(67-75) produced the maximum amount of crystallin precipitation in HLE from a >35-year-old human lens, whereas Pro-substituted αA-(66-80) peptide behaved like αA-(1-14) peptide with minimum protein precipitation. Under the experimental conditions about 5 µg of crystallins were precipitated per microgram of αA-(66-80) peptide in 24 h. Decreasing the hydrophobicity of αA-(66-80) peptide by substitution of a Val by Ala partially reduced the precipitation of crystallins. However, an 86% decrease in crystallin aggregation and precipitation was observed when we used a scrambled form of αA-(66-80) peptide (Figure S2 and Table 1).

**Table 1:** Peptides tested in this study for their crystallin aggregation properties.

Peptide	Sequence	Whether present in the lens	Amount of human lens proteins precipitated by the peptide (µg)
αA-(1-14)	CH3-MDVTIQHPWFKRTL	Yes	11
αA-(67-75)	DRDKFVIFL	Yes	142
αA-(66-75)	SDRDKFVIFL	Yes	44
αA-(66-80)	SDRDKFVIFLDVKHF	Yes	139
αA-(66-80-Pro)	SDRDKF**P**IFLDVKHF	No	14
αA-(66-80-Ala)	SDRDKF**A**IFLDVKHF	No	103
αA-(66-80-Scr)	FKISDHFKDVFRDVL	No	19

Water-soluble extract (2 mg) from 35-year-old human lens was incubated in 50 mM phosphate buffer, pH 7.2 at 37°C for 24 h in the presence or absence of various peptides (25 µg). The precipitate formed was collected by centrifugation at 1000× g for 10 min and dissolved in buffer containing 7 M urea and the amount of protein was estimated by Bio-Rad protein assay method. The values are an average of two independent experiments. Lens extract alone showed the precipitation of ∼12 µg of proteins after 24 h of incubation. Bold letters in sequence represent substitutions.

The data on crystallin aggregation and precipitation by αA-(66-80) peptide indicate that α-crystallin is the preferential target for the αA-(66-80) peptide, but that some precipitation of β- and γ-crystallin occurs as well (Figure 2C, lanes 2, 4,5, and 6 with protein bands >20 kDa). To determine whether β- and γ-crystallins precipitate in the absence of α-crystallin, we incubated HLE after removing α-crystallin by gel filtration. In the absence of α-crystallin, β- and γ-crystallins from a 70-year-old sample precipitated to a greater extent than the β- and γ-crystallins from 19-year-old sample (Figure S3). The greater precipitation of β- and γ-crystallins, in the absence of α-crystallin, in the aged HLE may be due to the interaction of αA-(66-80) with β- and γ-crystallins that have undergone age-related modifications.

### Hydrophobic interactions are involved in αA-(66-80)-induced HMW aggregate formation

**Figure 3:**
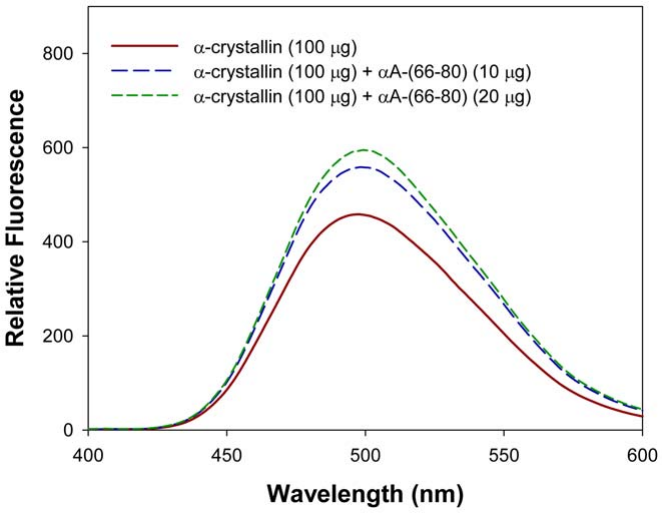
Bis-ANS binding to α-crystallin treated with or without αA-(66-80). α-Crystallin (100 µg) purified from a bovine lens extract was incubated with and without 10 or 20 µg of αA-(66-80) peptide in 50 mM phosphate buffer, pH 7.2 for 60 min and 10 µL of 20 mM bis-ANS prepared in 5% ethanol was added. Fluorescence was recorded after excitation at 390 nm in a Jasco FP750 spectrofluorometer. The fluorescence for αA-(66-80) was subtracted from the spectra for the complexes. These data show that the interaction of this peptide with α-crystallin leads to an overall increase in the hydrophobicity of the complex.

Hydrophobic interaction is presumed to be one of the major causes for the α-crystallin aggregation. Since the addition of αA-(66-80) peptide to α-crystallin results in aggregation of peptide–α-crystallin complex, we investigated whether peptide-induced enhancement of hydrophobicity plays a role by using the sensitive hydrophobic probe Bis-ANS to measure the overall hydrophobicity of proteins. As shown in Figure 3, binding of the αA-(66-80) peptide to α-crystallin increases the hydrophobicity of the α-crystallin–peptide complex. We found that the addition of αA-(66-80) peptide to α-crystallin results in ∼30% increase in Bis-ANS binding. The oligomeric size of the α-crystallin–peptide complex also increased (Figure 2B), suggesting that peptide-bound α-crystallin oligomeric size is far greater than the α-crystallin. However, the intrinsic Trp fluorescence of αA-crystallin did not show any shift in the emission maximum (data not shown), suggesting that the environment surrounding Trp residues is not affected when αA-(66-80) peptide interacts with the protein.

### αA-(66-80) peptide interaction with α-crystallin subunits and HMW aggregate formation

**Figure 4:**
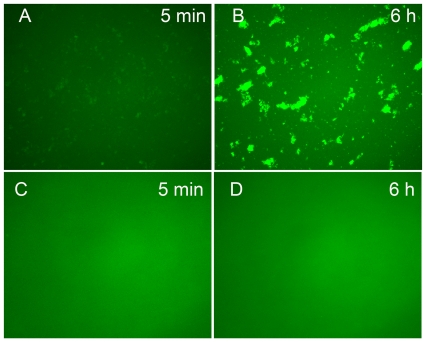
αA-(66-80) Peptide–induced HMW aggregate formation. Aggregates formed after incubation mixtures of 25 µg αA-(66-80) peptide with 200 µg α-crystallin, as described under methods, were re-suspended with Alexa-488 labelled αB-crystallin (αBT162C-488) in 50 mM phosphate buffer, pH 7.2, and the mixture was incubated at 37°C. The protein sample was removed after 5 min and 6 hrs and placed on a pre-cleaned glass slide and observed under the Leica fluorescence microscope using blue filter. The image was captured at 20× magnification. (A) Sample after 5 min; (B) sample after 6 h. As a control, a mixture of αA-(66-80-Pro) peptide–treated α-crystallin was incubated overnight and an aliquot was mixed with αBT162C-488 and observed under fluorescence microscope at 5 min (C) and 6 h D). The results show that α-crystallin aggregates formed after incubation with αA-(66-80) peptide bind αB-crystallin. The findings suggest that peptide- α-crystallin acts as a nucleus for binding of αB-crystallin.

To determine whether the α-crystallin–αA-(66-80) complex forms a nucleus for protein aggregation, αA-(66-80) peptide was added to α-crystallin and the aggregate formed was re-suspended with αB-crystallin that carried the T162C mutation [28], and the lone thiol group of the mutant was labeled with Alexa fluor 488 (αBT162C-488). The reaction mixture of αA-(66-80) peptide–α-crystallin aggregates and αBT162C-488 was incubated at 37°C for 6 h. Examination under the fluorescent microscope revealed that αBT162C-488 was clearly bound to αA-(66-80)-induced α-crystallin aggregates (Figure 4B), whereas such aggregation was not observed in samples containing Pro-substituted αA-(66-80) peptide (Figure 4D). Rather, homogenous distribution of Alexa-488 labeled αBT162C was observed. Under these experimental conditions, α-crystallin without peptide or αAT162C-488 showed a homogenous background without any fluorescence (Figure not shown). A time-lapse recording of the aggregation assay showed that the αBT162C-488 binding to αA-(66-80) peptide–α-crystallin complex begins soon after the incubation is started (Movie S1) and with time, leads to the formation of larger aggregates. Similarly, both β- and γ-crystallins were found to associate with the α-crystallin–αA-(66-80) peptide complex (Figures S4A and S4B) but it took nearly 24 h for the occurrence of significant binding of labeled β- or γ-crystallins to complexes of α-crystallin–αA-(66-80) peptide. WI protein from human lenses was re-suspended by sonication to obtain water-insoluble sonicate supernatant (WISS) and tested for its ability to interact with αBT162C-488. We found that the αBT162C-488 was attracted by the WISS only after the addition of αA-(66-80) peptide (Figure S5). WISS and αBT162C-488 did not interact in the presence of αA-(66-80-Pro) peptide to emit dense fluorescence from the aggregates (Figure S5C).

### Protein concentration affects αA-(66-80) peptide-induced aggregation of crystallins

**Figure 5:**
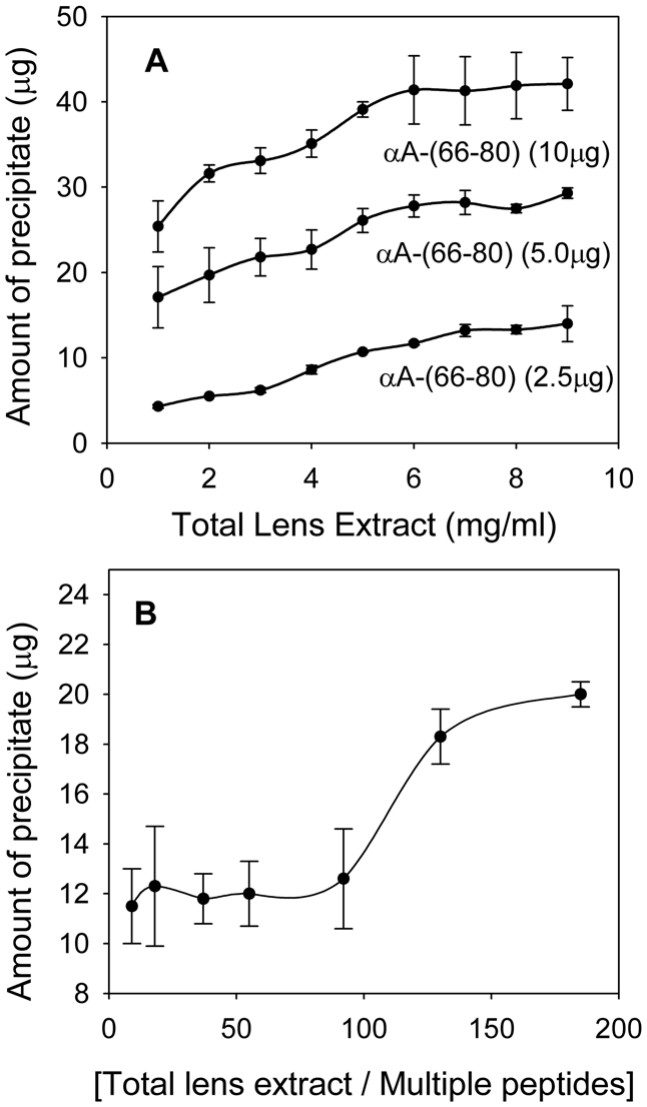
Effect of protein concentration on peptide-induced aggregation of human lens proteins. (A) Lens crystallin fraction (0.5–9 mg) was incubated with 2.5–10 µg of αA-(66-80) peptide in 1 ml buffer at 37°C. The amount of protein precipitated after 24 h was estimated using Bio-Rad protein assay reagent (n = 3). The amount of precipitate formed increased when the reaction mixture contained increased amounts of peptide or lens extract. (B) Effect of a mixture of peptides on lens proteins. Different concentrations of dialyzed human lens extract were mixed with a mixture of peptides consisting of αA-(66-80), αA-(66-75), αA-(67-75), αB-(1-18) and βA3/A1-(102-117), 1.0 µg each, to obtain 5- to 200-fold excess (by weight) of crystallins. The mixtures were incubated at 37°C for 24 h in 1 ml of 50 mM phosphate buffer, pH 7.2. The precipitate formed was collected by centrifugation and estimated as above. The results show that the presence of several peptides in low concentration is sufficient to induce lens crystallin precipitation when the crystallin concentration is high. Therefore, an *in vivo* lens protein concentration of 400 mg/ml is likely to aggregate and precipitate even when the peptide concentration is 100's of times lower than that of crystallins.

The crystallin concentration in human lens is estimated to be ∼400 mg/ml [29] and of this amount, ∼160 mg/ml is α-crystallin. Therefore, to determine the effect of a high *in vivo* concentration of lens crystallins on peptide-induced aggregation of lens proteins, αA-(66-80) peptide was incubated at 37°C with 100 to 4000-fold (w/w) excess WS crystallins from 35-year-old human lenses. The amount of protein precipitated was measured after 24 h of incubation. With a mixture of 5 µg of αA-(66-80) peptide and 1 mg of lens proteins, in an 18∶1 molar ratio of α-crystallin-to-αA-(66-80) peptide, ∼17 µg of protein precipitated from the reaction mixture (∼20 kDa monomer weight for α-crystallin used in calculation). The amount of precipitate increased to ∼40 µg when the peptide concentration in the incubation mixture was 2-fold higher (Figure 5A graph with 10 µg peptide) and the crystallin concentration was 5-fold greater. We found that the amount of precipitate formed gradually increased with increasing peptide or crystallin concentrations in the reaction mixture (Figure 5A). Examination of the precipitates under electron microscopy showed that interaction of αA-(66-80) peptide with α-crystallin or lens proteins produced amorphous aggregates (Figure S6 F and H).

### Cumulative effects of different α-crystallin peptides together in the lens

Most of the aggregation-inducing peptides in the lens are found in the nuclear region of aged and cataract lenses (Figure 1). We determined the cumulative effects of αA-(66-80), αA-(66-75), αA-(67-75), αB-(1-18), and βA3/A1-(102-117) peptides, which are all present in aged and cataract human lenses. A peptide mixture, 5.0 µg, of equal amounts of the above-mentioned peptides was incubated for 24 h (37°C) with various amounts of soluble protein isolated from 65-year-old human lenses. After 24 h of incubation of the reaction mixture, we discovered that a 1∶200 ratio of peptide mixture to crystallins yielded an amount of precipitation comparable to that observed with 5 µg of αA-(66-80) peptide and 2 mg of crystallin (Figure 5A and 5B). Figure 5B also shows that the peptide-induced aggregation and precipitation of lens crystallin increased dramatically with peptide-to-crystallin ratios of greater than 1∶100 (w/w).

### Effect of αA-(66-80) peptide on the chaperone activity of α-crystallin

**Figure 6:**
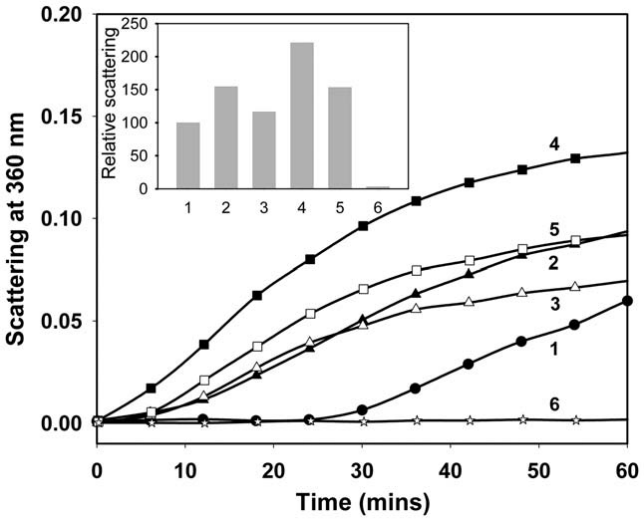
The αA-(66-80) peptide functions as an anti-chaperone. The effect of αA-(66-80) peptide on the chaperone activity of αB-crystallin against denaturing ADH (150 µg) was examined using the assay procedure described earlier [10]. (1) ADH; (2) ADH+αA-(66-80) (25 µg); (3) ADH+αB (30 µg)+αA-(66-80) (25 µg); (4) ADH+αA-(66-80) (50 µg); (5) ADH+αB (30 µg)+αA-(66-80) (50 µg); and (6) ADH+αB (30 µg). The relative scattering by the samples at the 40-min time point is shown in the inset graph. As expected, ADH aggregation is suppressed in the presence of αA-crystallin. But ADH aggregation is increased in presence of αA-(66-80) peptide, suggesting that the peptide facilitates the aggregation of denaturing ADH. Additionally, the chaperone activity of αB-crystallin is diminished in reactions containing αA-(66-80) peptide, suggesting that the peptide interferes in the chaperone activity of αB-crystallin that exhibits anti-chaperone activity.

After demonstrating in HLE the ability of the αA-(66-80) peptide to precipitate α-crystallin from the WS fraction, we investigated whether αA-(66-80) peptide interaction affects the chaperone activity of α-crystallin and αB-crystallin using ADH aggregation assay. (Because it is known that both αA- and αB-subunits display comparable chaperone activity as homo-oligomers and hetero-oligomers, only αB-crystallin was tested in this study.) As shown in Figure 6, αA-(66-80) incubation with αB-crystallin resulted in a loss of αB-crystallin chaperone activity. Furthermore, we observed that the presence of αA-(66-80) peptide in the absence of chaperone proteins enhanced the aggregation of denaturing ADH (Figure 6). Saturating concentrations of αA-(66-80) peptide not only nullified the chaperone activity of αB-crystallin but also enhanced the aggregation of proteins in the assay. As with αB-crystallin, purified bovine lens α-crystallin also exhibited loss of chaperone activity when αA-(66-80) peptide was used in chaperone assays (Figure S7). The loss of α-crystallin chaperone activity was proportional to the amount of the peptide in the reaction mixture. When the reaction mixtures were observed under EM, only amorphous protein aggregates were seen with ADH, ADH + αA-(66-80), ADH + α-crystallin + αA-(66-80) (Figure S6 A-C) and ADH + αB-crystallin + αA-(66-80) peptide (Figure not shown).

### αA-(66-80) Peptide and its β-sheet configuration forms β-amyloid fibril-like structures

**Figure 7:**
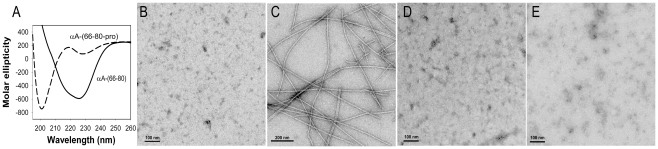
Amyloid-like characteristics of the αA-(66-80) peptide. (A) Far-UV CD spectra of 0.1 mg/ml αA-(66-80) peptide and the Pro-substituted peptide in phosphate buffer, pH 7.2, obtained as described under methods. Proline substitution abolishes the β-sheet structure of the peptide. (B, C, D) TEM micrographs of peptides incubated in 50 mM phosphate buffer (pH 7.2) at 37°C. (B) αA-(66-80) at 0 min; (C) αA-(66-80) after 24 h; and (D) TEM of the Pro-substituted αA-(66-80) peptide after 24 h incubation. (E) TEM of the αA-(1-14) peptide after 24 h incubation. TEM studies show that αA-(66-80) peptide forms fibrils (D), whereas following Pro-substitution, the same peptide is unable to form fibrils (E), suggesting that a propensity to form β-sheet structure is necessary for fibril formation. The αA-(1-14) peptide did not form fibrils (E) and did not show β-sheet structure (data not shown).

Because αA-(66-80) peptide has a β-amyloid signature sequence [22], we investigated whether this peptide has properties similar to those of β-amyloid. Indeed, the αA-(66-80) peptide exhibits the characteristic β-sheet structure (Figure 7A) when analyzed with a far-UV (ultraviolet) CD spectropolarimeter. Spectral ellipticity was lost when one of the Val was replaced by Pro to create the peptide SDRDKF**P**IFLDVKHF, suggesting that the presence of a β-sheet breaker Pro disrupts the structure of the peptide. We also found that thioflavin and Congo Red dyes interact with αA-(66-80) peptide and show a time-dependent increase in fluorescence (data not shown). The binding of these two dyes to proteins indicates the presence of both pre-amyloid-like and amyloid-like structures. Indeed, amyloid fibril-like structures were found when we examined by TEM αA-(66-80) incubated for 24 h at 37°C in phosphate buffer (pH 7.2), (Figure 7C). At 0 time, the αA-(66-80) sample had no fibril-like structures (Figure 7B). As expected, the Pro-substituted αA-(66-80) peptide completely lost its ability to form fibrils (Figure 7D). The control peptide αA-(1-14) did not show any fibril-like structures during a 24-h incubation period (Figure 7E). Incubation of αA-(66-80) peptide with α-crystallin resulted in complete suppression of fibril formation by the peptide and only aggregates of peptide and α-crystallin was seen (Figure S6F).

## Discussion

Human lenses ≥50 years old apparently have a negligible amount of WS α-crystallin in the nuclear region [16], [17]. The reasons for the partitioning of WS α-crystallin into the WI fraction over time are not known, but this event is believed to contribute to the formation of age-related aggregates that accumulate in the center of the lens. Aged and age-related cataract human lenses with nuclear opacity show an increased amount of crystallin fragments as well as aggregated proteins as compared to the young lenses [9]. Our studies of the peptide distribution in aged and cataract human lenses show that the WIS fraction has significantly more peptides than the WS fraction [5], [10]. The hyperbaric oxygen (HBO)-induced cataract model, designed to mimic the aging human lens, shows that lens opacity begins developing in the nuclear region [30]. The nuclear region of HBO-treated guinea pig lens contains higher levels of crystallin fragments than the outer cortical region (unpublished data). Previously we reported that peptides from aged and cataract human lenses have ∼4-fold greater light scattering activity [10]. Based on the current findings we believe that a nexus exists between crystallin breakdown, crystallin-derived peptide accumulation, crystallin aggregation and the development of age-related nuclear cataract. To investigate further the role of crystallin-derived peptides in lens protein aggregation and insolubilization of α-crystallin in the nuclear region of aging lenses, we first determined the distribution of several αA-crystallin–derived peptides in human lens fractions. Mass spectrometric analysis and identification of peptides isolated from human lenses showed that several of the peptides from αA-crystallin are present only in the nuclear region (Figure 1A). Mass spectrometric imaging of human lens cross sections has also shown that a number of crystallin-derived peptides are present in the nuclear region of human lens and that a concentration gradient for the peptides exists in which the innermost region of the lens has the highest concentration of the peptides whereas the outermost region has the lowest amount of peptides [5], [11]. Of the peptides identified from the nuclear region, αA-(66-80) is the most prominent member and it consists of residues 70-80 in αA-crystallin that contribute to the chaperone site [20]. We found that the amount of αA-(66-80) peptide present in >70-year-old human lenses is 2.33±0.6 nmol/g lens (or 7.28±1.8 nmol/g lens protein if we assume that 32% of lens weight is protein [1]). This value is significantly higher than the highest amount of lens β-amyloid (Aβ, 1.37 nmol/g lens protein) found in lenses from Alzheimer's disease (AD) patients [31] or the amount of Aβ in brain tissue of AD patients (0.01±0.001 nmol/g soluble protein and 0.22±0.01 nmol/g insoluble protein) [32]. In addition to αA-(66-80) peptide, we also estimated that the WIS fraction of >70-year-old human lens also contains 0.55±0.03 nmol αA-(66-75), 1.4±0.1 nmol of αA-(67-80), 0.33±0.02 nmol αA-(67-75) per g lens tissue, all rising from 66-80 region of αA-crystallin and capable of precipitating α-crystallin.

At this time it is not known how αA-(66-80) peptide is generated in aging lenses since we cannot attribute the action of a specific lens protease to the generation of the peptide. The αA-(66-75), αA-(66-80) and αA-(67-75) peptides are likely to be generated from αA-(66-80) peptide by the action of aminopeptidases and carboxypeptidases in the lens [33], [34]. It is unclear why only a few peptides from a given crystallin are present in the cortical and nuclear regions of the lens (Figure 1A) while the rest of the peptides generated during proteolysis of crystallins are completely degraded into amino acids. We hypothesize that binding of the peptides to other crystallins may be protecting them from the action of peptidases. Further studies are underway to confirm this.

The data on crystallin aggregation and precipitation (Figures 2, S1 and S2) by αA-(66-80) peptide indicate that α-crystallin is the preferential target for the αA-(66-80) peptide, but that some precipitation of β- and γ-crystallin occurs as well. In the absence of α-crystallin, β- and γ-crystallins from 74-year-old HLE precipitated to a greater extent than β- and γ-crystallins from 19-year-old HLE (Figure S3). The heightened precipitation of β-and γ-crystallins, in the absence of α-crystallin, in the aged HLE may be due to the interaction of αA-(66-80) with β- and γ-crystallins that have undergone age-related modifications. Studies are needed to confirm this possibility and to identify the αA-(66-80) interaction sites in precipitating crystallins. The data indicate that the peptides αA-(66-80), αA-(66-75) and αA-(67-80), along with αB-(1-18) [10] and βA3/A1-(59-74) [35], might be involved in the aggregation and insolubilization of α-crystallin in the nuclear region of human lenses during aging. α-Crystallin aggregation and complete insolubilization in the nuclear region of aged lens was first reported in 1976 [16], but the mechanisms underlying aggregation and precipitation remain unknown. It is yet to be determined which other peptides among >25 peptides present in aged human lenses [10] also interact with crystallins, induce aggregation and precipitation.

Based on our earlier study [36] and the data presented here, it appears that binding of the αA-(66-80) peptide to α-crystallin increases the hydrophobicity of the α-crystallin–peptide complex (Figure 3), with a concomitant gain of function, leading to the additional binding of α-, β- and γ-crystallins to form HMW complexes (Figures 2B, 4 and S5) that precipitate rapidly. We believe that the peptide binds to α-crystallin subunits, at multiple sites, as in the case of βA3/A1-(102-117) peptide binding to αB-crystallin [35], to bring about conformational change sufficient to increase the overall hydrophobicity of the complex above that of native protein. An increase in the net attractive forces between hydrophobic complexes formed by αA-(66-80) peptide and a decrease in the stability of these complexes are likely involved in aggregation and precipitation of crystallins in complex with peptides found in aging lenses.

The inability of Pro-substituted αA-(66-80) peptide to precipitate α-crystallin from HLE and the decreased precipitation of α-crystallin with Ala-substituted αA-(66-80) peptide (Figure 2A and Table 1) suggest that the propensity to form β-sheet structure and relative hydrophobicity are important attributes of a peptide involved in α-crystallin insolubilization. In addition, the aggregation-inducing activity of αA-(66-80) peptide appears to be sequence specific because αA-(66-80-scr) (a peptide having the scrambled αA-(66-80) peptide sequence) was unable to precipitate α-crystallin from the lens extract (Figure S2 and Table 1). The sequence specificity is further highlighted by the inability of hydrophobic β-sheet-forming αA-(70-88) peptide to precipitate α-crystallin from lens extracts. The precipitation of α-crystallin present in CLE and HLE by αA-(67-75) and αA-(66-75) (Figure 2A and Table 1) shows that more than one peptide generated from αA-(66-80) region of aging human lenses has the ability to cause α-crystallin aggregation and precipitation and that the minimum sequence required for this action is αA-(67-75).

Depletion of α-crystallin from HLE or CLE in response to the addition of αA-(66-80) peptide was proportional to the amount of peptide added to HLE or CLE when a fixed concentration of lens extract was used (Figures 2B and S2). However, when an increasing amount of CLE was used, increased precipitation of the α-crystallin–peptide complex occurred (Figure 5A), and when the peptide-to-HLE ratio was over 100, there was an exponential increase in the precipitation of crystallins from HLE (Figure 5B), suggesting that a low concentration of αA-(66-80) peptide or a mixture of peptides having similar properties is sufficient to bring about significant precipitation of α-crystallin from HLE. The lowest αA-(66-80) peptide concentration (2.5 µg/ml) that caused lens protein precipitation during incubation (Figure 5A) was less than the *in vivo* αA-(66-80) peptide concentration confined to the nuclear region of a 72-year-old human lens (Figure 1C). Since 1 µg of αA-(66-80) peptide alone has the potential to precipitate 5.6 µg of lens proteins in 24 h when the lens protein concentration is 2 mg/ml (Table 1), and since crystallin precipitation increases with increased HLE concentrations, we can predict that the combined action of several peptides *in vivo* would result in the precipitation of several hundred micrograms of crystallins. The αA-(66-80) peptide was 5-fold more effective in precipitating crystallins compared to the cytosolic protein precipitation activity of β-amyloid [23]. β-Amyloid as well as β-sheet-forming proteins are known to cause aggregation of cellular proteins [24], [37] and this process is implicated in protein aggregation diseases. The possibility that crystallin aggregation and precipitation occurs from the combined action of crystallins and peptides is underscored by the fact that the aging lens has several peptides capable of precipitating α-crystallin [10] and the peptides have a cumulative effect on crystallin aggregation and precipitation (Figure 5B) are primarily confined to the lens nuclear region (Figure 1), whose volume is a fraction of estimated lens volume of ∼225 µl. Further, the peptides accumulate *in vivo* for several years and to decades, thereby providing ample time to interact and cause precipitation. We found that αA-(66-80) peptide starts to accumulate as early as 18 years of age. By 70 years of age, we found that the amount of αA-(66-80) peptide is increased at least 7-fold as compared to the amount in young lenses (Figure 1C). The interaction of the peptide with α-crystallin appears to result in the attraction of additional crystallin molecules (Figure 4B) in a crowded environment to form larger aggregates that precipitate with time. This view is supported by data from Alexa-labeled αB-, β- and γ-crystallins and time-lapse experiments, which demonstrated that incubation with the αA-(66-80) peptide induces the formation of HMW crystallin aggregates (Figures 4B, S4 and S5).

It is known that the molecular crowding effect on protein aggregation is non-linear and that as the protein concentration increases, the aggregation increases exponentially [38]. Based on our observations of increasing lens crystallin precipitation with increasing amounts of crystallin extract and a mixture of αA-(66-80), αA-(66-75), αA-(67-75), αB-(1-18) and βA3/A1-(102-117) (Figure 5B), we believe that the presence of several different peptides at low concentrations *in vivo* is sufficient to precipitate a significant portion of lens crystallins and cause light scatter, as seen in aged cataract lenses. We and others estimate that the degradation of crystallins in aged lenses is as much as 10 to 20 percent of the total lens proteins [9], [10], [39]. Of the degraded crystallins, those that are <4 kDa will be present in a concentration of 0.8 to 1.2 mg per lens [10]. We estimate that crystallin aggregation activity in merely 10 percent of the ∼1 mg peptides in a lens [10] would be equivalent to the interaction of 100 µg of peptides with ∼40 mg α-crystallin in a confined lens volume of <225 µl. A conservative estimate of precipitation in this scenario is >500 µg of proteins. We know from numerous *in vitro* experiments that precipitation of ∼25 µg protein is sufficient to scatter light. Additionally, from the data in Figure S3, we reason that age-related modifications increase the susceptibility of β- and γ-crystallins to precipitation. Taken together, the results of our study suggest that age-related accumulation of crystallin-derived peptides and their interaction with other crystallins in a volume-excluding environment may be responsible for the partitioning of α-crystallins into WIS fraction in lenses over 40 years of age. Such partitioning of α-crystallins was first reported in 1976 [16] but what underlies such age-related changes remained unknown till now.

In previous studies, we demonstrated the existence of anti-chaperone activity in crystallin fragments prepared *in vitro* from oxidized lens proteins and in a βA3/A1-crystallin fragment from aged lenses [27], [35]. However, our demonstration in the current study of αA-(66-80) anti-chaperone activity in the WIS fraction of lens extracts is particularly important because the αA-(66-80) peptide is found in aged and cataract lenses and is derived from αA-crystallin, a small heat shock protein [sHSP] chaperone whose function is critical for maintaining lens transparency [2].

Amyloid-like fibrils have been noted to form when α, β and γ-crystallins are incubated at 60°C in the presence of 1 M guanidine hydrochloride (GnHCl) or under acidic conditions [40]. Other investigators also found that αA-(70-88) peptide aggregates into amyloid fibril-like structures when incubated at pH 2.0 or after exposure to 1 M GnHCl or after vigorous shaking at 60°C [41]. Our study is the first observation of fibril formation by a peptide from αA-crystallin under physiological pH conditions *in vitro*. We propose that the 70-80 region in αA-crystallin is responsible for the formation of amyloid-like fibrils from αA-crystallin.

### Proposed mechanism for peptide-induced lens aging and cataract development

**Figure 8:**
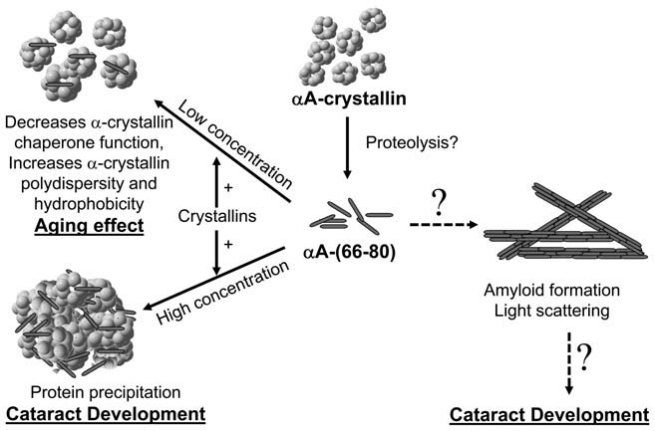
Schematic of αA-(66-80) peptide–mediated lens aging and cataract development. *In vivo* αA-(66-80) peptides interact with crystallins and affect the structure and organization of crystallins, leading to age-related changes and cataract development. At low concentrations, αA-(66-80) specifically interacts with α-crystallin, resulting in increased hydrophobicity and polydispersity and decreased chaperone activity of α-crystallin. At higher concentrations, αA-(66-80) binds to crystallins, leading to insolubilization of crystallins and cataract development. Localized accumulation of the αA-(66-80) peptide has the potential to form light-scattering amyloid-like structures. However, the latter is less likely because the interaction of the peptide with crystallins suppresses the formation of fibrils.

We found that a correlation exists between crystallin aggregate formation *in vivo* during lens aging and αA-(66-80) peptide-induced aggregation of soluble lens crystallins *in vitro*. Therefore, we suggest that αA-(66-80) peptides in the lens play an integral role in lens aging and cataract development. Based on our data, we propose a mechanism for peptide-induced lens aging (Figure 8). For an as yet unknown reason, αA-(66-80) peptide is released from αA-crystallin. The peptide preferentially interacts with α-crystallin. At low concentrations, αA-(66-80) induces a conformational change in α-crystallin, increasing the hydrophobicity of α-crystallin, decreasing its chaperone-like function, and enhancing the association between α-crystallin subunits, all characteristics of α-crystallins in aged lenses. At higher concentrations, αA-(66-80) causes the precipitation of α-crystallins and other proteins, which manifest as a cataract. It is difficult to estimate the amount of αA-(66-80) required for cataract formation, as other anti-chaperone peptides and post-translational modifications can also contribute to cataract development, as has been shown with HBO-induced cataract model. It should also be noted that our study of the effects of αA-(66-80) revealed that β- and γ-crystallins from 70-year-old human lenses, known to have undergone increased oxidation and modifications, precipitate to a greater extent than the β- and γ-crystallins from 19-year-old lenses when same amount of the peptide is used (Figure S2).

We did not find any peptide *in vivo* that can counteract the effects of the αA-(66-80) peptide. Rather, we found additional peptides—αA-(66-75), αA-(67-75), αA-(67-80), αB-(1-18), and βA3/A1-(102-117)—in the lens with properties similar to those of the αA-(66-80) peptide. While the αA-(66-80) peptide has the propensity to form fibrils *in vitro*, it is not apt to form similar structures *in vivo* because α-crystallin would most likely sequester these peptides and the effective concentration of the free peptide would be kept low (Figure 8). In support of this possibility, we observed that only HMW aggregates were formed when the αA-(66-80) peptide was incubated with α-crystallin or HLE (Figure S6 F and H).

Taken together, the results of this study reveal a novel mechanism for age-related crystallin aggregation and the formation of cataracts. Known for many years as one of the least understood protein conformational diseases, cataracts are a major cause of severe visual impairment and blindness in an estimated 20 million people worldwide. This study demonstrates a number of important observations that expand our understanding of cataract formation: 1) The αA-(66-80) peptide generated in human lenses interacts with α-crystallin and causes its aggregation and precipitation, as seen in aging and cataract lenses; 2) the αA-(66-80) peptide binds to denaturing β- and γ-crystallins, facilitating their aggregation; 3) the interaction of αA-(66-80) peptide with α-crystallin renders the interaction site as a nucleation site that attracts α-, β- and γ-crystallins to form HMW aggregates, 4) a high protein concentration (molecular crowding) leads to increased peptide-induced aggregation and precipitation of lens crystallins, and 5) the αA-(66-80) peptide suppresses the chaperone activity of α-crystallins. Our identification of the αA-(66-80) peptide as one of the causative agents of lens protein aggregation begins a new path toward understanding the mechanisms for age-related cataractogenesis.

## Materials and Methods

### Synthetic peptides

The peptides used in this study were obtained from GenScript Corporation (Piscataway, NJ), and the University of Missouri Structural Biology Core Facility. The purity level of the synthetic peptides exceeded 95%. The peptides (2 mg) were dissolved in 1 ml of sterile water. Peptides that were not readily soluble were briefly sonicated to solubilize them. The samples were then filtered through a 0.2 µm filter, and the concentration of the peptide was estimated using the micro-BCA protein assay kit (Thermo Scientific). The peptide incubations and assays were carried out in phosphate buffer (50 mM sodium phosphate buffer containing 150 mM NaCl, pH 7.2), unless otherwise mentioned. The AQUA peptide, SDRDKFV(IC^13^N^15^)FLDVKHF, (>96% purity) was obtained from Sigma-Genosys. Synthetic peptides were used in binding, aggregation and precipitation studies.

### Isolation of low molecular weight (LMW) peptides from human lenses

Human donor lenses were obtained from the Heartland Lions Eye Bank (Columbia, MO) and kept at −70°C until use. Cataract lenses were obtained as a kind gift from the Patney Eye Clinic (Rajkot, India) and were stored frozen at −70°C until use. Provisions of the Declaration of Helsinki for research on human tissue were followed while collecting the lenses. Indian cataract lenses used in the study were of type 2, as described by Pirie [42]. The lenses were thawed on ice, and the capsules were carefully removed with a forceps. To isolate the water-soluble-free (WSF) peptides, the lenses were homogenized in 6.0 ml of homogenizing buffer (phosphate buffer saline, pH 7.4, containing 2 mM Tris(2-carboxyethyl) phosphine hydrochloride (Thermo Scientific) and 5 µl of protease inhibitor mixture and then centrifuged at 16000× g for 1 h. The supernatant was then passed through a 10 kDa cut-off filter (Millipore) to obtain the WSF peptides. The retentate of the 10 kDa cut-off filter was treated with homogenizing buffer containing 6 M urea to dissociate peptides bound to proteins and was then centrifuged, as above, to collect the water-soluble–bound (WSB) peptides. The WIS fraction was treated with 6 ml of homogenizing buffer containing 6 M urea and sonicated on ice for 30 sec, followed by centrifugation and 10 kDa filtration to isolate WIS peptides. To isolate the cortical and nuclear peptides, the lenses were stirred on ice, using a micro-stir bar, in 3 ml of homogenizing buffer containing 6 M urea until the cortex had dissolved (about 3 min). The nuclear fractions of the lens were then transferred to a fresh tube and homogenized with 6 ml of the same buffer. Both the cortex and the nuclear fractions were then processed, as described above, to collect the LMW peptides. Peptides isolated from human lenses were used in identification/quantification studies.

### Determination of αA-(66-80) peptide concentration in the aged lens

AQUA peptide (1 nmol) was dissolved in 100 µl of a 10% (v/v) formic acid solution. Two stock solutions of AQUA peptide (0.4 pmol/µl and 4 pmol/µl) were prepared in acetonitrile/water/90% formic acid buffer (30∶69∶1). Different amounts of AQUA peptide (0–40 pmol) were spiked into 150 µl of LMW peptide fractions. The samples were desalted using PepClean C18 columns (Thermo Scientific). The bound peptides were eluted using 70% acetonitrile containing 0.1% formic acid. For mass spectrometry analysis, 5 µl of the sample was added to 5 µl of alpha-cyano-4-hydroxycinnamic acid, and 0.5 µl of the mixture was applied to the sample target and analyzed with a 4700 MALDI TOF/TOF mass spectrometer (Applied Biosystems Inc.). The amount of αA-(66-80) present in the lens was calculated from the AQUA peptide standard graph.

### Protein aggregation analysis

WI aggregates were separated from the lens extract and peptide incubations by centrifugation at 3000 rpm for 10 min. The soluble fraction was injected into a TSK G5000PW_XL_ (Tosoh Bioscience) gel filtration column connected to a high-performance liquid chromatography (HPLC) (Shimadzu, Columbia, MD) coupled to multi-angle light- scattering and dynamic light-scattering detectors (Wyatt Technology, Santa Barbara, CA). The amount of protein that remained soluble in the incubation mixture and the molecular mass were determined using ASTRA software (Wyatt Technology). The WIS aggregates from the incubation mixtures were analyzed by SDS-PAGE.

### αA-(66-80) peptide in HMW aggregate formation

αA-(66-80) Peptide (25 µg) was added to 200 µg of α-crystallin in phosphate buffer (0.2 ml of 50 mM, pH 7.2) and the mixture was incubated at 37°C. After overnight incubation, a visible aggregate was observed in α-crystallin samples with peptides, whereas no visible precipitate was seen in α-crystallin without peptides. The aggregate was collected by centrifugation and the pellet was re-suspended with a cysteine-substituted αB-crystallin labeled with Alexa fluor 488 (αBT162C-488). The single cysteine residue of αBT162C was labeled with a thiol reactive Alexa fluor 488 dye, as per the protocol of the manufacturer (Molecular Probes). The reaction mixture was incubated further at 37°C. The protein sample was removed at 5 min, 6 h, and 24 h and placed on the pre-cleaned glass slide and observed under the fluorescence microscope using blue filter. The image was captured at 20× magnification in a Leica microscope. As a control, a mixture of αA-(66-80–Pro) peptide–treated α-crystallin+αBT162C labeled with Alexa fluor 488 was used. The binding of β- and γ-crystallins to peptide-induced α-crystallin aggregates was investigated by the addition of Alexa fluor 488–labeled β- or γ-crystallins in place of αBT162C-488.

### Peptide-induced aggregation of lens crystallins

Human lenses (65 years old) were homogenized in phosphate buffer containing 6 M urea. The urea-soluble supernatant was collected by centrifugation of the homogenate at 15000× g for 1 h. The supernatant was dialyzed (10 kDa) against phosphate buffer to remove urea and LMW peptides. The dialyzed sample was used as such or after passing it through a Superdex G200 column to collect the α-crystallin fraction. Different amounts of total lens extract or α-crystallin were incubated, with or without peptide(s), at 37°C in 1 ml phosphate buffer. At the end of incubation the tubes were centrifuged at 15000× g for 20 min to collect the insoluble aggregates. The pellet was solubilized in 6 M urea and the amount of protein was estimated using Bio-Rad protein assay reagent. The amount of protein precipitates in control tubes was subtracted from the precipitates in peptide-treated tubes to estimate the peptide-induced protein precipitation.

### Time-lapse recording of αA-(66-80) peptide-induced HMW aggregate formation

αBT162C-488 was added to α-crystallin–αA-(66-80) peptide aggregate, as above, and an aliquot was taken in a glass slide with a well. The sample was photographed in an Olympus IX70 inverted microscope every minute for 5 h. The images were compressed into a movie using MetaMorph software (Molecular Devices, Inc., Sunnyvale, CA).

### Circular dichroism (CD) spectroscopy

The αA-(66-80) peptide (0.1 mg/ml in phosphate buffer) was examined immediately after preparation. CD spectra were acquired with a Jasco J-815 CD spectropolarimeter (Jasco Inc., Easton, MD) using a 2.0-mm cuvette at 25°C. The spectrum derived represents an average of six scans.

### Transmission electron microscopy (TEM) study

The morphology of amyloid-like fibril formation by the αA-(66-80) peptide was studied by TEM (Jeol, Ltd., Tokyo, Japan). Briefly, the αA-(66-80) peptide (1 mg/ml) was suspended in phosphate buffer and incubated at 37°C for different time intervals, ranging from 0 h to 24 h. Aliquots (5 µl) were applied to carbon-coated, Formvar-filmed 400 mesh copper grids at room temperature and left for 1 min. The excess solution was then wicked away with filter paper. The peptides on the grid were stained with 5 µl of 2% (w/v) uranyl acetate solution for 1 min. This solution was then wicked off, and the grid was air-dried and then examined using a JEOL 1400 TEM (120 kV). TEM was operated at 80 kV. The images were obtained using digital imaging software from Gatan Digital Micrograph, (Gatan, Inc., Warrendale, PA).

## Supporting Information

Figure S1 **Effect of different concentrations of the αA-(66-80) peptide on the calf lens extract (CLE).** CLE, 200 µg, was incubated with different amounts of αA-(66-80) in 50 mM phosphate buffer, pH 7.2 at 37°C for 16 h. The total incubation volume was 155 µl. After incubation, the samples were centrifuged and 100 µl of the supernatant was analyzed in a multi-angle light scattering (MALS) system. Peaks of α, βH, βL and γ-crystallin are marked. The inset graph shows the percentage of soluble lens proteins recovered after incubation with the peptides. The results show that the interaction of αA-(66-80) with the CLE results in selective removal of α-crystallin.
(TIF)

Figure S2 **Comparison of crystallin aggregation inducing activity of αA-(66-80) peptide and a peptide having the scrambled αA-(66-80) sequence, αA-(66-80-scr).** The peptides were incubated with CLE as described in Figure 2 and the precipitate formed was removed by centrifugation and the soluble proteins were analyzed adopting the procedure described in legend for Figure 2. The results show that scrambled peptide of αA-(66-80) has negligible α-crystallin precipitation activity.
(TIF)

Figure S3 **Effect of the αA-(66-80) peptide on 19- and 70-year-old HLE that lacked α-crystallin.** The α-crystallin fraction was removed from HLE by gel filtration. The β- and γ-crystallin fractions were pooled and incubated with 50 µg of the αA-(66-80) peptide in phosphate buffer. After 16 h at 37°C, the reaction mixture was centrifuged, and the soluble fraction was analyzed by multi-angle light scattering. The amount of protein that remained soluble at the end of incubation is depicted in the figure. The results show that, in the absence of α-crystallin, β- and γ-crystallins interact with the αA-(66-80) peptide, and this interaction leads to the precipitation of the complex. In addition, crystallins from aged lenses appeared more susceptible to precipitation by the peptide.
(TIF)

Figure S4 **αA-(66-80) Peptide–induced aggregation of crystallins to form HMW aggregates.** Visible aggregates from the overnight incubation of αA-(66-80) peptide (25 µg) and α-crystallin (200 µg) in phosphate buffer (50 mM, pH 7.2) at 37°C were re-suspended in Alexa fluor 488–labeled β- (A) or γ-crystallins (B) and incubated further. The protein sample was removed at 5 min, 6 h and 24 h and placed on a pre-cleaned glass slide and observed under the fluorescence microscope using a blue filter. The image was captured at 20× magnification. In the absence of αA-(66-80) peptide, fluorescently labeled β- and γ-crystallin show no association with α-crystallin for 24 h (panel a). Both β- and γ-crystallins, albeit slowly when compared to α-crystallin (Fig. 3), were incorporated into αA-(66-80) peptide–α-crystallin aggregates (panels b, c, and d). Based on these data we propose that *in vivo* interaction of αA-(66-80) peptide may be responsible, at least in part, for the formation of HMW aggregates composed of α-, β- and γ-crystallins.
(TIF)

Figure S5 **The αA-(66-80) peptide recruits proteins to HMW aggregates.** The αA-(66-80) peptide (25 µg) was added to WISS proteins (200 µg) from human lens in phosphate buffer (50 mM, pH 7.2). The mixture was incubated at 37°C overnight. The tubes were centrifuged to remove soluble-free peptides. The insoluble aggregates were incubated further at 37°C for 6 h with Alexa fluor 488–labeled αB-crystallin (αBT162C-488). At the end of incubation, the sample was observed under fluorescence microscope, as described earlier. A. WISS + αBT162C-488; B. WISS + αA-(66-80) + αBT162C-488; C. WISS + αA-(66-80-pro) + αBT162C-488. The results show that in about 6 h, αBT162C-488 binds to WISS treated with αA-(66-80) peptide, whereas such an interaction does not occur in the absence of αA-(66-80) peptide or with samples treated with αA-(66-80-Pro) peptide. Based on these data, we propose that *in vivo* interaction of αA-(66-80) peptide may be responsible, at least in part, for the formation of HMW aggregates.
(TIF)

Figure S6 **Suppression of αA-(66-80) peptide fibril formation by ADH and crystallins.** TEM micrographs of proteins and peptides following aggregation reactions. Protocol for TEM is described under methods. (A) ADH (250 µg); (B) ADH (250 µg) + αA-(66-80) peptide (25 µg); (C) ADH (250 µg) + α-crystallin (100 µg) + αA-(66-80) peptide (25 µg); and (D) αA-(66-80) peptide (1 mg/ml) incubated at 37°C for 24 h. A, B and C represent samples from Figure S7. Experiment details are included in the legend for Figure S7. (D) Sample was prepared as described in legend for Figure 7. (E-H) Samples were incubated in 50 mM phosphate buffer (pH 7.2) at 37°C for 24 h and then examined under TEM. (E) α-Crystallin (1 mg/ml); (F) αA-crystallin (0.2 mg) + αA-(66-80) peptide (25 µg); (G) HLE (1 mg/ml); and (H) HLE (1 mg/ml) + αA-(66-80) peptide (25 µg). The results show that αA-(66-80) peptide does not form fibrils in the presence of ADH, αA-crystallin or HLE that has structure similar to that of fibrils formed by the peptide.
(TIF)

Figure S7 **Anti-chaperone activity of the αA-(66-80) peptide against bovine α-crystallin in the ADH aggregation assay.** Aggregation assay was carried out, as described earlier [10], after incubation of α-crystallin with the peptide for 35 min at 37°C. The results show that the αA-(66-80) peptide is capable of suppressing the chaperone activity of α-crystallin.
(TIF)

Movie S1 **Time-lapse recording of binding of αBT162C-488 to αA-(66-80) peptide-induced aggregates of α-crystallin**
(MOV)
