# Supplementary figures and images for: αA-Crystallin Peptide 66 SDRDKFVIFLDVKHF 80 Accumulating in Aging Lens Impairs the Function of α-Crystallin and Induces Lens Protein Aggregation
Source: PLoS One. 2011 Apr 28;6(4):e19291. doi: 10.1371/journal.pone.0019291 (PMC3084282; doi:10.1371/journal.pone.0019291)

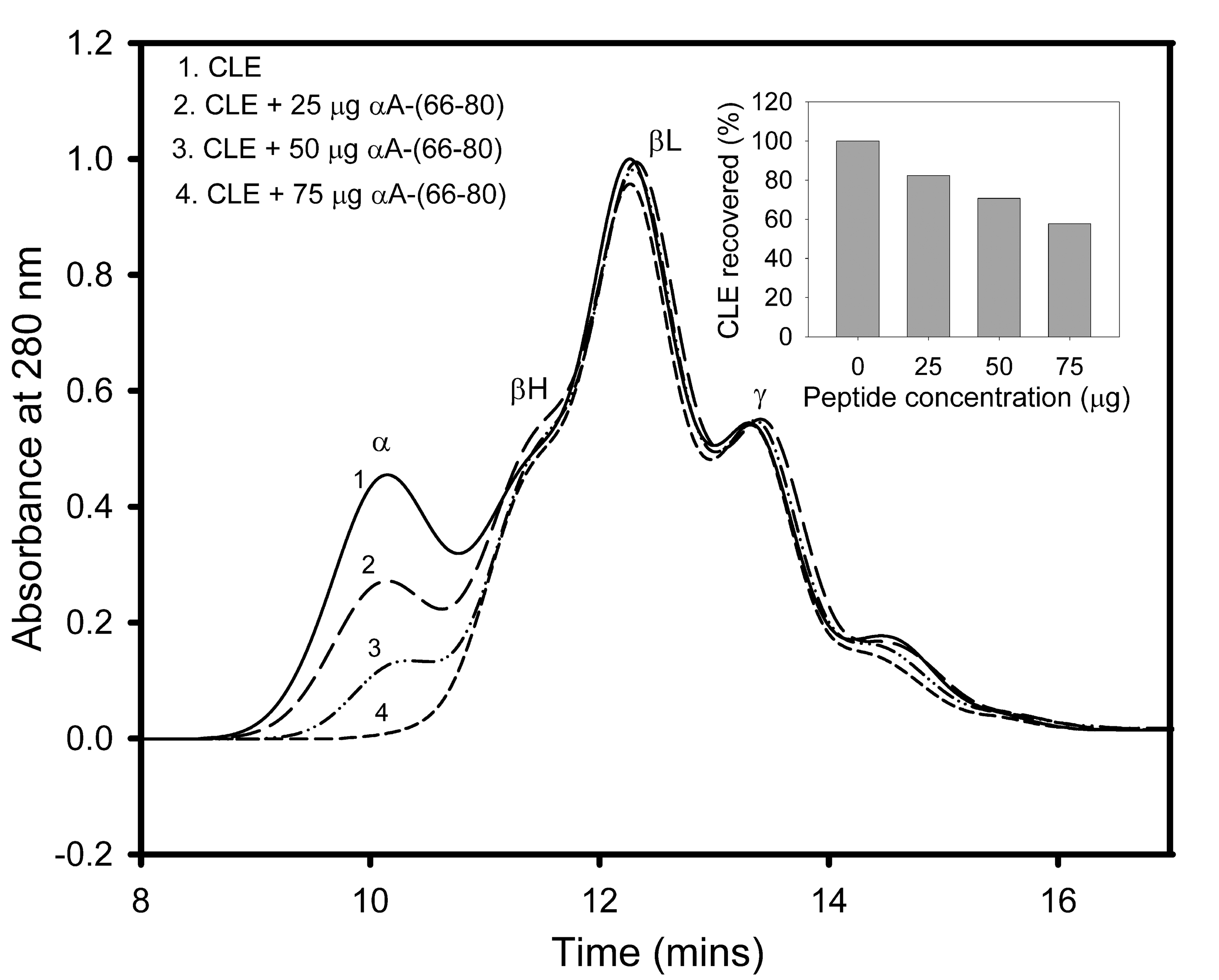

Supplement: Figure S1 — Effect of different concentrations of the αA-(66-80) peptide on the calf lens extract (CLE). CLE, 200 µg, was incubated with different amounts of αA-(66-80) in 50 mM phosphate buffer, pH 7.2 at 37°C for 16 h. The total incubation volume was 155 µl. After incubation, the samples were centrifuged and 100 µl of the supernatant was analyzed in a multi-angle light scattering (MALS) system. Peaks of α, βH, βL and γ-crystallin are marked. The inset graph shows the percentage of soluble lens proteins recovered after incubation with the peptides. The results show that the interaction of αA-(66-80) with the CLE results in selective removal of α-crystallin. (TIF) [file pone.0019291.s001.tif]

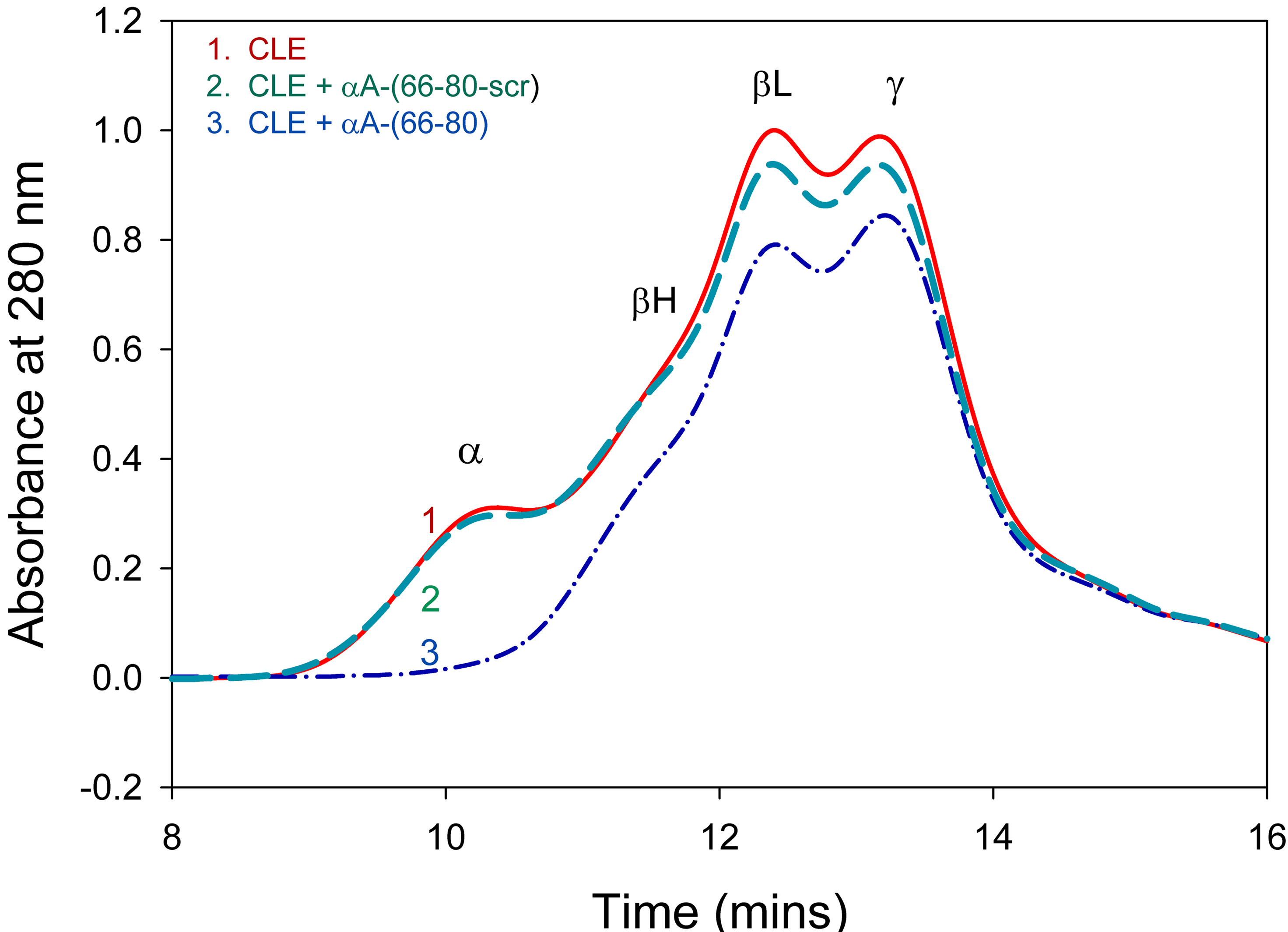

Supplement: Figure S2 — Comparison of crystallin aggregation inducing activity of αA-(66-80) peptide and a peptide having the scrambled αA-(66-80) sequence, αA-(66-80-scr). The peptides were incubated with CLE as described in Figure 2 and the precipitate formed was removed by centrifugation and the soluble proteins were analyzed adopting the procedure described in legend for Figure 2. The results show that scrambled peptide of αA-(66-80) has negligible α-crystallin precipitation activity. (TIF) [file pone.0019291.s002.tif]

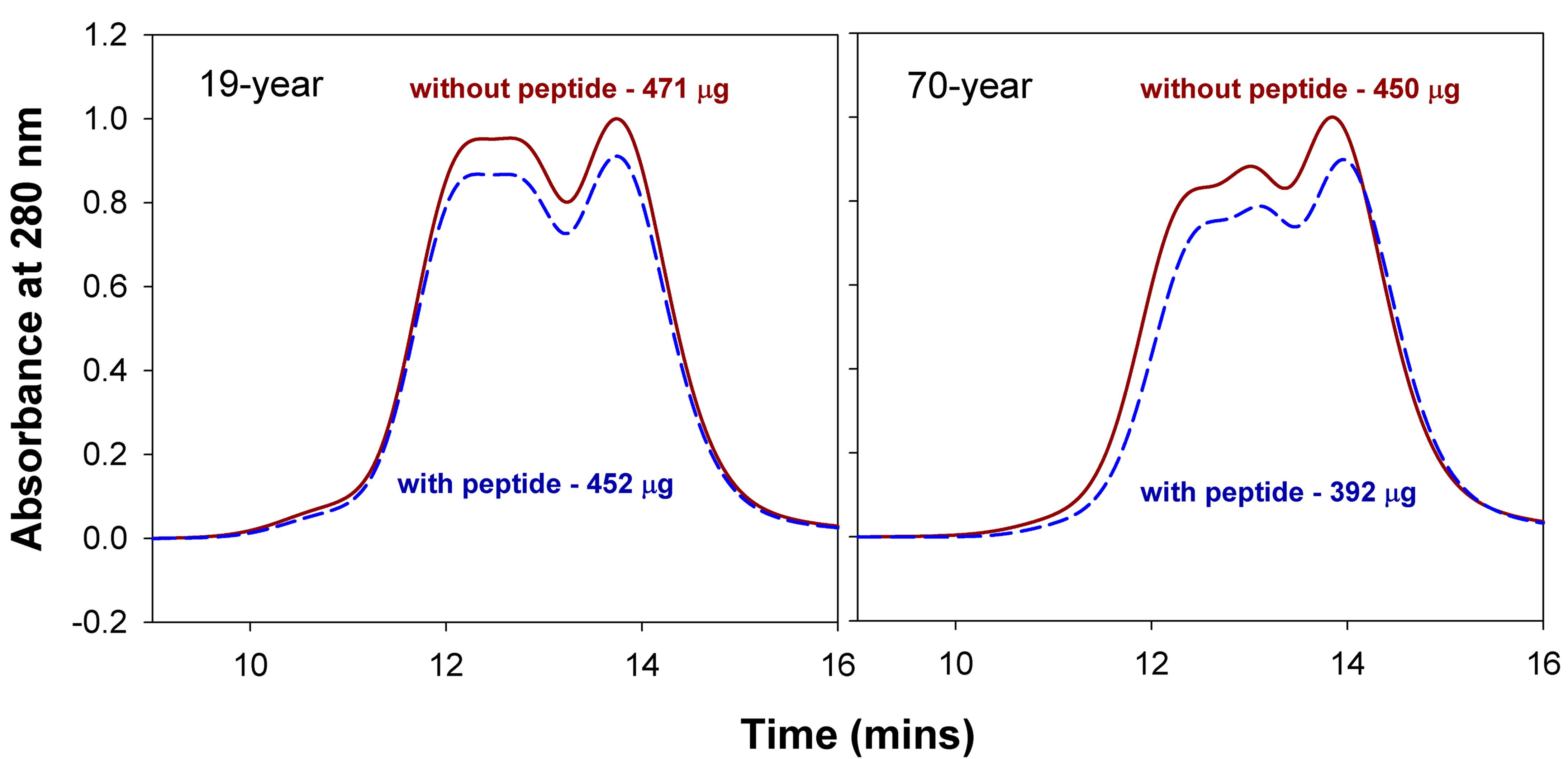

Supplement: Figure S3 — Effect of the αA-(66-80) peptide on 19- and 70-year-old HLE that lacked α-crystallin. The α-crystallin fraction was removed from HLE by gel filtration. The β- and γ-crystallin fractions were pooled and incubated with 50 µg of the αA-(66-80) peptide in phosphate buffer. After 16 h at 37°C, the reaction mixture was centrifuged, and the soluble fraction was analyzed by multi-angle light scattering. The amount of protein that remained soluble at the end of incubation is depicted in the figure. The results show that, in the absence of α-crystallin, β- and γ-crystallins interact with the αA-(66-80) peptide, and this interaction leads to the precipitation of the complex. In addition, crystallins from aged lenses appeared more susceptible to precipitation by the peptide. (TIF) [file pone.0019291.s003.tif]

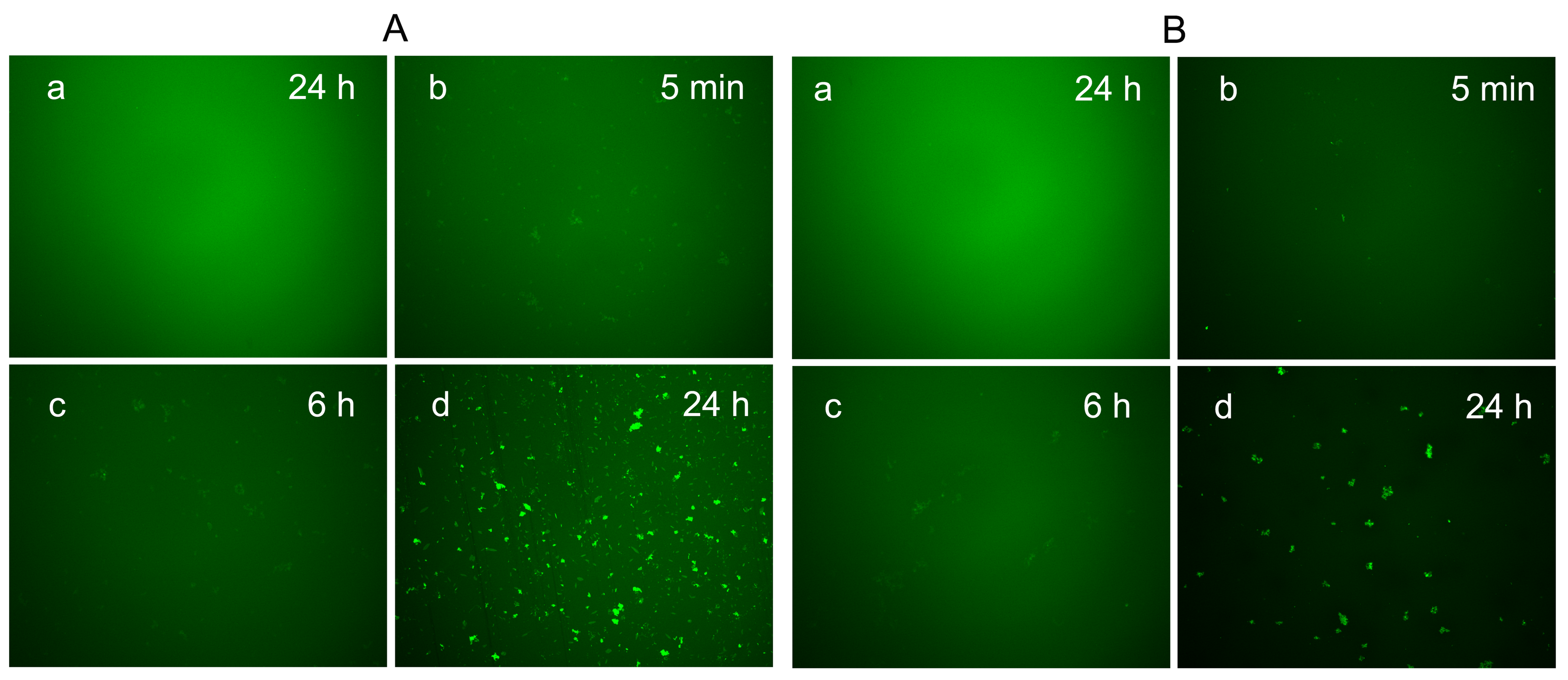

Supplement: Figure S4 — αA-(66-80) Peptide–induced aggregation of crystallins to form HMW aggregates. Visible aggregates from the overnight incubation of αA-(66-80) peptide (25 µg) and α-crystallin (200 µg) in phosphate buffer (50 mM, pH 7.2) at 37°C were re-suspended in Alexa fluor 488–labeled β- (A) or γ-crystallins (B) and incubated further. The protein sample was removed at 5 min, 6 h and 24 h and placed on a pre-cleaned glass slide and observed under the fluorescence microscope using a blue filter. The image was captured at 20× magnification. In the absence of αA-(66-80) peptide, fluorescently labeled β- and γ-crystallin show no association with α-crystallin for 24 h (panel a). Both β- and γ-crystallins, albeit slowly when compared to α-crystallin (Fig. 3), were incorporated into αA-(66-80) peptide–α-crystallin aggregates (panels b, c, and d). Based on these data we propose that in vivo interaction of αA-(66-80) peptide may be responsible, at least in part, for the formation of HMW aggregates composed of α-, β- and γ-crystallins. (TIF) [file pone.0019291.s004.tif]

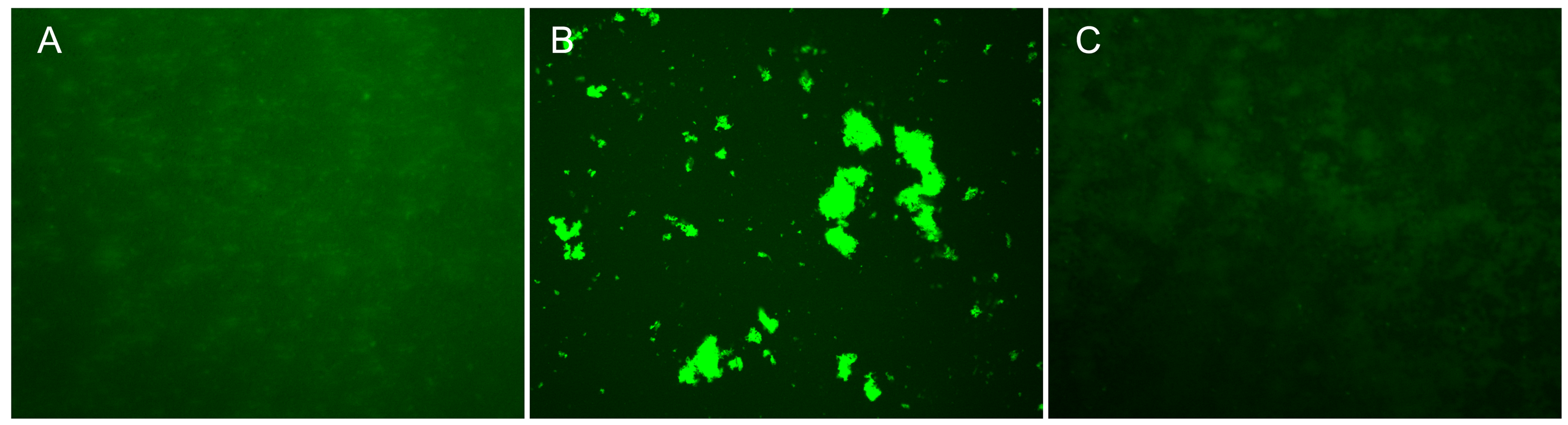

Supplement: Figure S5 — The αA-(66-80) peptide recruits proteins to HMW aggregates. The αA-(66-80) peptide (25 µg) was added to WISS proteins (200 µg) from human lens in phosphate buffer (50 mM, pH 7.2). The mixture was incubated at 37°C overnight. The tubes were centrifuged to remove soluble-free peptides. The insoluble aggregates were incubated further at 37°C for 6 h with Alexa fluor 488–labeled αB-crystallin (αBT162C-488). At the end of incubation, the sample was observed under fluorescence microscope, as described earlier. A. WISS + αBT162C-488; B. WISS + αA-(66-80) + αBT162C-488; C. WISS + αA-(66-80-pro) + αBT162C-488. The results show that in about 6 h, αBT162C-488 binds to WISS treated with αA-(66-80) peptide, whereas such an interaction does not occur in the absence of αA-(66-80) peptide or with samples treated with αA-(66-80-Pro) peptide. Based on these data, we propose that in vivo interaction of αA-(66-80) peptide may be responsible, at least in part, for the formation of HMW aggregates. (TIF) [file pone.0019291.s005.tif]

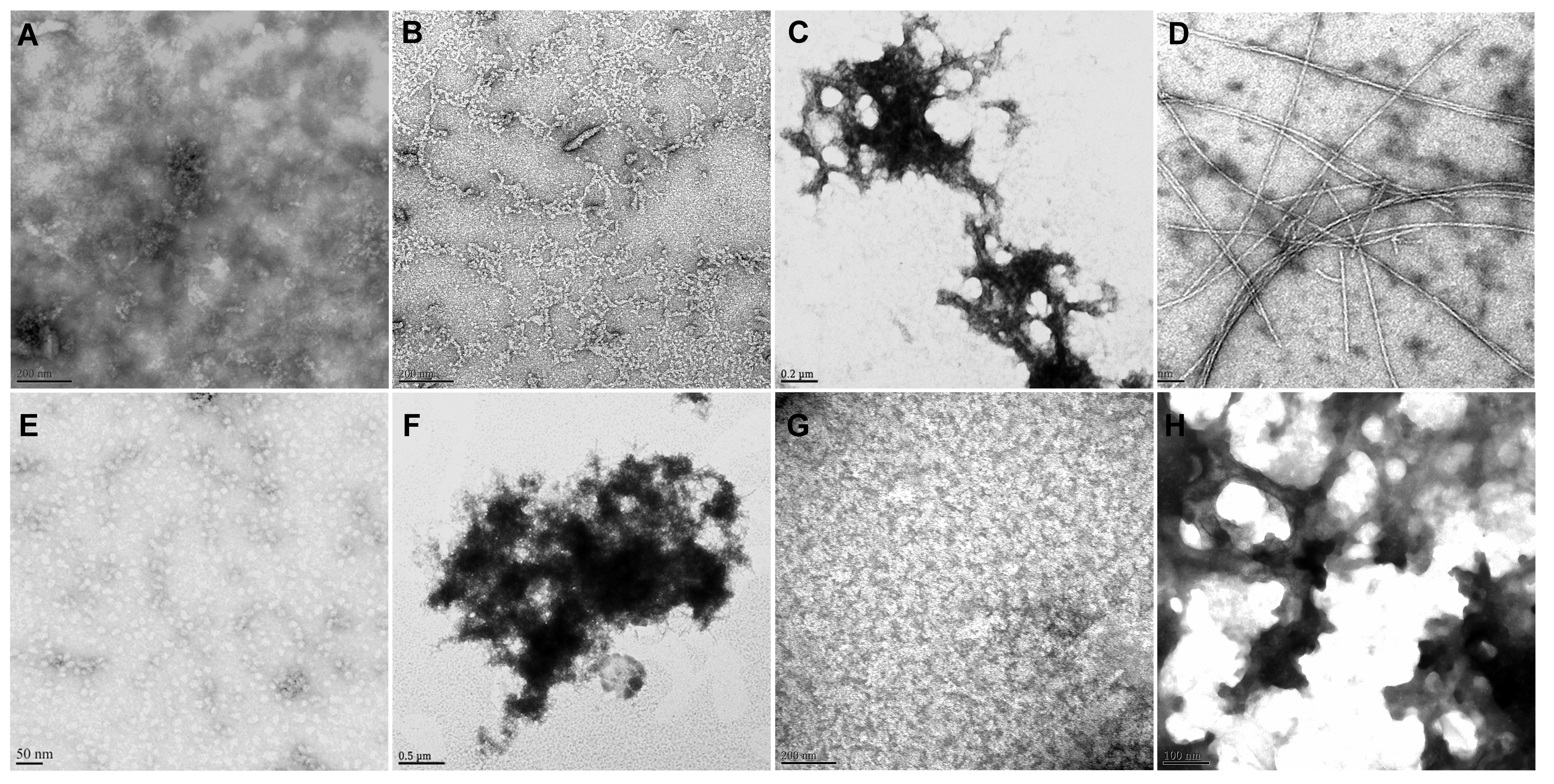

Supplement: Figure S6 — Suppression of αA-(66-80) peptide fibril formation by ADH and crystallins. TEM micrographs of proteins and peptides following aggregation reactions. Protocol for TEM is described under methods. (A) ADH (250 µg); (B) ADH (250 µg) + αA-(66-80) peptide (25 µg); (C) ADH (250 µg) + α-crystallin (100 µg) + αA-(66-80) peptide (25 µg); and (D) αA-(66-80) peptide (1 mg/ml) incubated at 37°C for 24 h. A, B and C represent samples from Figure S7. Experiment details are included in the legend for Figure S7. (D) Sample was prepared as described in legend for Figure 7. (E-H) Samples were incubated in 50 mM phosphate buffer (pH 7.2) at 37°C for 24 h and then examined under TEM. (E) α-Crystallin (1 mg/ml); (F) αA-crystallin (0.2 mg) + αA-(66-80) peptide (25 µg); (G) HLE (1 mg/ml); and (H) HLE (1 mg/ml) + αA-(66-80) peptide (25 µg). The results show that αA-(66-80) peptide does not form fibrils in the presence of ADH, αA-crystallin or HLE that has structure similar to that of fibrils formed by the peptide. (TIF) [file pone.0019291.s006.tif]

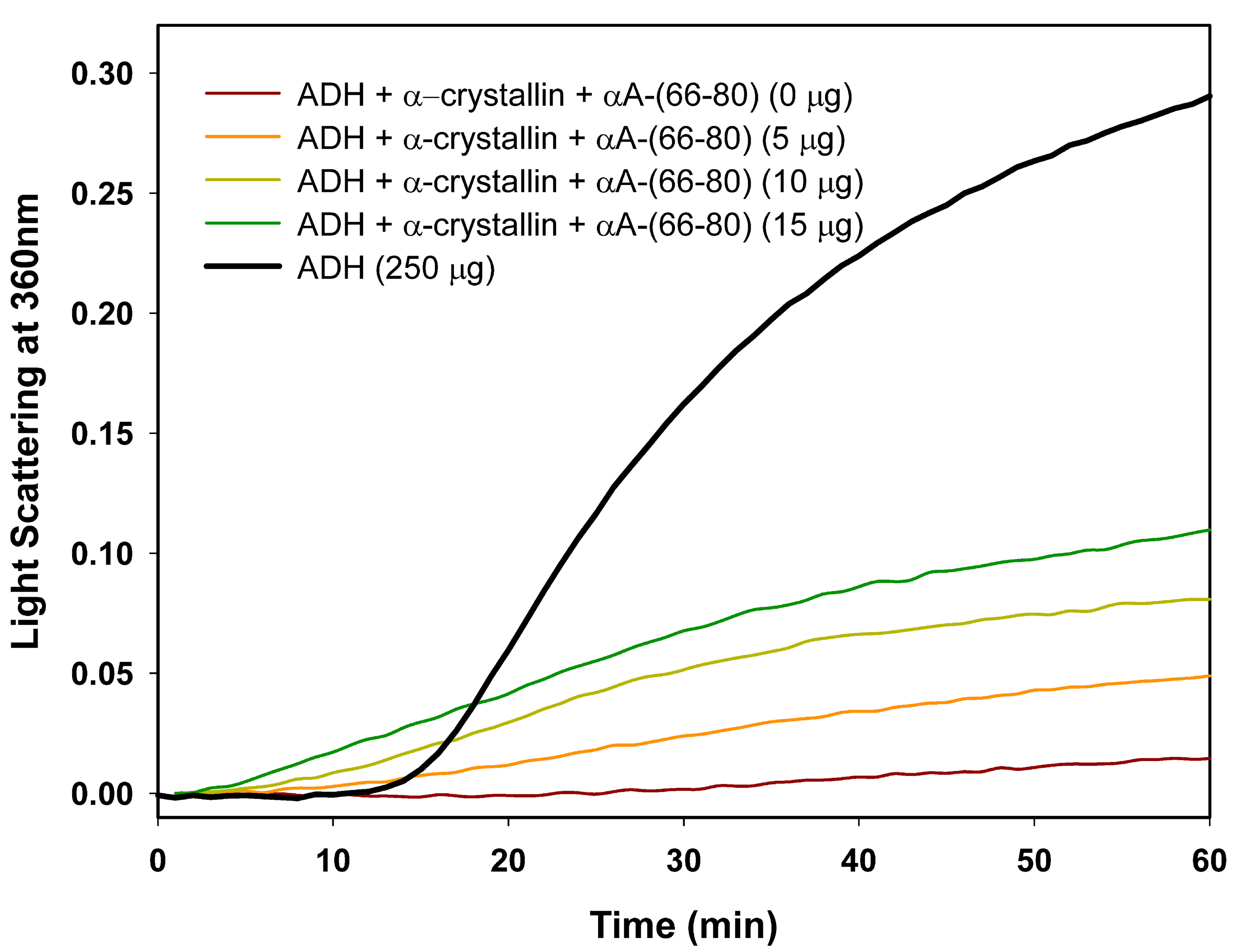

Supplement: Figure S7 — Anti-chaperone activity of the αA-(66-80) peptide against bovine α-crystallin in the ADH aggregation assay. Aggregation assay was carried out, as described earlier [10], after incubation of α-crystallin with the peptide for 35 min at 37°C. The results show that the αA-(66-80) peptide is capable of suppressing the chaperone activity of α-crystallin. (TIF) [file pone.0019291.s007.tif]
